# Physiological response and proteomics analysis of *Reaumuria soongorica* under salt stress

**DOI:** 10.1038/s41598-022-06502-2

**Published:** 2022-02-15

**Authors:** Shipeng Yan, Peifang Chong, Ming Zhao, Hongmei Liu

**Affiliations:** 1 College of Forestry, Gansu Agricultural University, Lanzhou, 730070 China; 2 Gansu Province Academy of Qilian Water Resource Conservation Forests Research Institute, Zhangye, 734000 China

**Keywords:** Biological techniques, Ecology, Physiology, Plant sciences, Systems biology

## Abstract

Soil salinity can severely restrict plant growth. Yet *Reaumuria soongorica* can tolerate salinity well. However, large-scale proteomic studies of this plant’s response to salinity have yet to reported. Here, *R. soongorica* seedlings (4 months old) were used in an experiment where NaCl solutions simulated levels of soil salinity stress. The fresh weight, root/shoot ratio, leaf relative conductivity, proline content, and total leaf area of *R. soongorica* under CK (0 mM NaCl), low (200 mM NaCl), and high (500 mM NaCl) salt stress were determined. The results showed that the proline content of leaves was positively correlated with salt concentration. With greater salinity, the plant fresh weight, root/shoot ratio, and total leaf area increased initially but then decreased, and vice-versa for the relative electrical conductivity of leaves. Using iTRAQ proteomic sequencing, 47 177 136 differentially expressed proteins (DEPs) were identified in low-salt versus CK, high-salt versus control, and high-salt versus low-salt comparisons, respectively. A total of 72 DEPs were further screened from the comparison groupings, of which 34 DEPs increased and 38 DEPs decreased in abundance. These DEPs are mainly involved in translation, ribosomal structure, and biogenesis. Finally, 21 key DEPs (SCORE value ≥ 60 points) were identified as potential targets for salt tolerance of *R. soongolica*. By comparing the protein structure of treated versus CK leaves under salt stress, we revealed the key candidate genes underpinning *R. soongolica*’s salt tolerance ability. This works provides fresh insight into its physiological adaptation strategy and molecular regulatory network, and a molecular basis for enhancing its breeding, under salt stress conditions.

## Introduction

Soil salinization is one of the main environmental factors limiting the sustainable development of agriculture and forestry worldwide^1^. Salinization impairs the productive potential of soil, destroys the habitat of plants, reduces the diversity of communities, and disrupts the ecological chain, which leads to the degradation or loss of ecosystem functions. According to recent statistics, the total area of saline-alkali land globally has reached 954 million hectares, with an annual expansion rate of 10%^2,3^. Especially at risk are arid and semi-arid areas, where climate conditions characterized by little precipitation and strong evaporation further promote the accumulation of salt in the soil surface. Therefore, it is imperative that researchers cultivate new varieties of salt-tolerant plants and use more salt-tolerant plants in regional cultivation.

To cope with the ion toxicity and osmotic stress caused by salt stress, plants have evolved a suite of adaptive mechanisms to minimize such damage to cells and tissues^4^. These mainly consist of morphological adaptations^5^, regulation of osmotic substances^6^, defensive functioning of the antioxidant enzyme system^7^, changes in the photorespiration pathway^8^, and ion zone isolation in cells^9^. In addition, some salt-secreting plants can form salt glands and use them or vesicles to secrete excess salt out of the body to avoid a large accumulation of salt ions^10^. In recent years, with rapid advances in proteomics technology, the proteomics approach has provided a powerful shortcut to predict the response of plants to salt stress conditions. Using proteomics technology to reveal differences in protein expression of plants under salt stress is now a research hotspot in the post-genomic era. Previous studies have analyzed the protein composition and change of cell and subcellular structures in different tissues and organs of salt-stressed plants, such as in Lobular^11^, okra^12^, rice^13^, alfalfa^14^, and licorice^15^, thereby uncovering many salt-reactive proteins conferring salt stress-resistance traits. These include aquaporins, ribosomal protein, heat shock proteins protein kinases, ornithine decarboxylase, ascorbate peroxidase as well as some transcription factors, with another study showing that proteins related to alkaloid synthesis could play a major role in the production of plant secondary metabolites^16^.

*Reaumuria soongorica* is a typical perennial salt-yielding halophytic shrub, which shows strong adaptability to saline and desert soils, and is a representative dominant species in salt-alkali desert areas of grassland ecosystems. This species plays a crucial role in stabilizing shifting sands, thereby helping to prevent soil erosion and desertification^17^. Further, the *R. soongorica* community provides a good pasture in desert areas^18^, with excellent soil improvement effects^19^. Therefore, this plant species offers an ideal material for studying and analyzing the physiological responses and molecular regulation mechanism underpinning salt tolerance. To date, studies on salt tolerance of *R. soongorica* have mainly focused on its physiological and biochemical indexes, such as ion absorption, seed germination, and antioxidant capacity of callus^17,20,21^, with no reports yet on the effects of salt stress on its proteomics. In this study, NaCl was used to impose a salt stress treatment, and a soil salt simulation experiment was conducted to measure the physiological indexes of *R. soongorica* seedlings. Meanwhile, label-free technology was used to study the effects of salt stress on the types and variation of differentially expressed proteins in *R. soongorica* seedlings, with the aim of mining those proteins related to salt stress. Clarifying the related metabolic pathways involved in the salt tolerance process of *R. soongorica* can provide important clues for further elucidating the physiological and molecular response mechanisms of *R. soongorica* plants to salt stress conditions, and this should provide a more effective scientific basis for breeding enhanced salt tolerant traits in *R. soongorica*.

## Results

### Effects of NaCl concentrations on growth indicators of *R. soongorica* seedlings

As shown in Table 1, when compared with control A (i.e., 0 mM NaCl), both the fresh weight and root/shoot ratio of *R. soongorica* in group B (i.e., 200 mM NaCl) were significantly higher. However, both fresh weight and root/shoot ratio gradually decreased in group C (i.e., 500 mM NaCl). When the NaCl concentration reached that of group C (i.e., 500 mM NaCl), the growth of *R. soongorica* was significantly inhibited. The fresh weight of above-ground and root tissues was respectively 43.82% and 50.99% that of the control, and these differences were significant (P < 0.05). Under the NaCl treatment with the B concentration, the water content of the above-ground and root tissues, as well as the total leaf area of leaves, exceeded that of the control. However, when the NaCl concentration was C, the water content of above-ground and root tissues were significantly lower than those of the control.

**Table 1:** Effect of different NaCl concentration treatments on fresh weight, ratio of root, water content and total leaves area of *R. soongorica* plants.

NaCl concentration (mM L^−1^)	Fresh weight (mg)		Fresh weight percentage (%)		Dry weight (%)	Water content of plant (%)			Total leaves area of plant (cm^2^)
	Above-ground	Root	Above-ground	Root	Root-shoot ratio	Above-ground		Root	
A	331.67 ± 21.08b	134.67 ± 6.81b	100.00	100.00	0.49 ± 0.06b	75.20 ± 3.58ab		64.47 ± 2.48a	9.66 ± 0.21b
B	392.00 ± 23.52a	154.67 ± 11.24a	118.19	114.85	0.60 ± 0.03a	79.68 ± 2.10a		69.12 ± 2.05a	10.75 ± 0.69a
C	145.33 ± 13.50c	68.67 ± 7.51c	43.82	50.99	0.41 ± 0.03c	71.90 ± 3.08b		53.27 ± 2.07b	5.55 ± 0.51c

**Figure 1:**
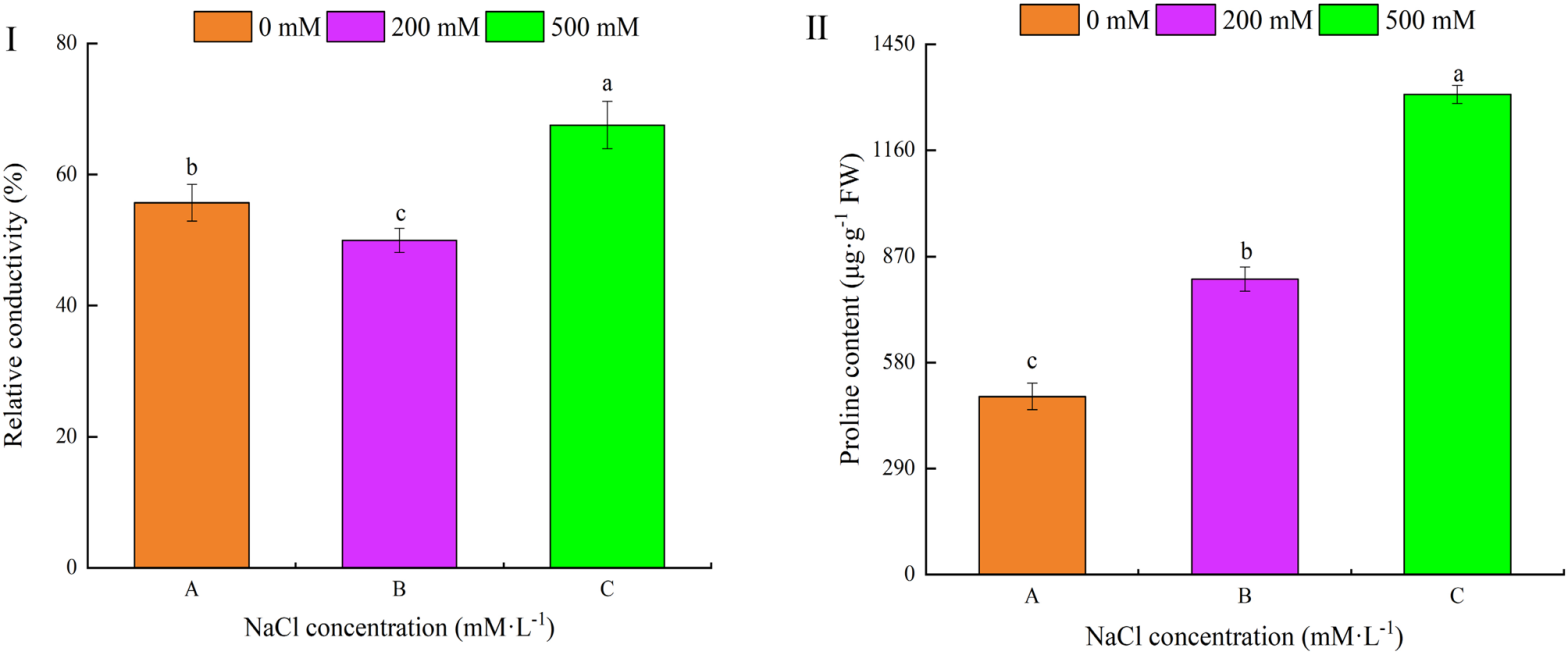
Effects of NaCl concentrations on the relative conductivity and proline content of *R. soongorica* leaves. I: relative conductivity II: proline content.

Means of 10 replicates ± S.D. are shown. Values followed by different letters differ significantly according to Duncan’s multiple range tests at P < 0.05. Different lowercase letters indicate that each index is significantly different at different salt concentrations. The same as Fig. 1.

### Effects of NaCl concentrations on relative conductivity and proline content of *R. soongorica* leaves

Under salt stress, Fig. 1-I shows the responsive changes in the relative conductivity and proline content of *R. soongorica* leaves. The relative conductivity decreased at first and then increased with an increasing NaCl stress concentration, and the differences were statistically significant. Meanwhile, leaf proline content also increased significantly (Fig. 1-II). These results indicated that *R. soongorica* could adapt to a salt stress environment by adjusting its leaf-level proline content. Under low salt stress, the cell membrane system of *R. soongorica* leaves was not damaged by stress, and its cell membrane had strong stability and could adequately adapt to a certain salt environment.

**Figure 2:**
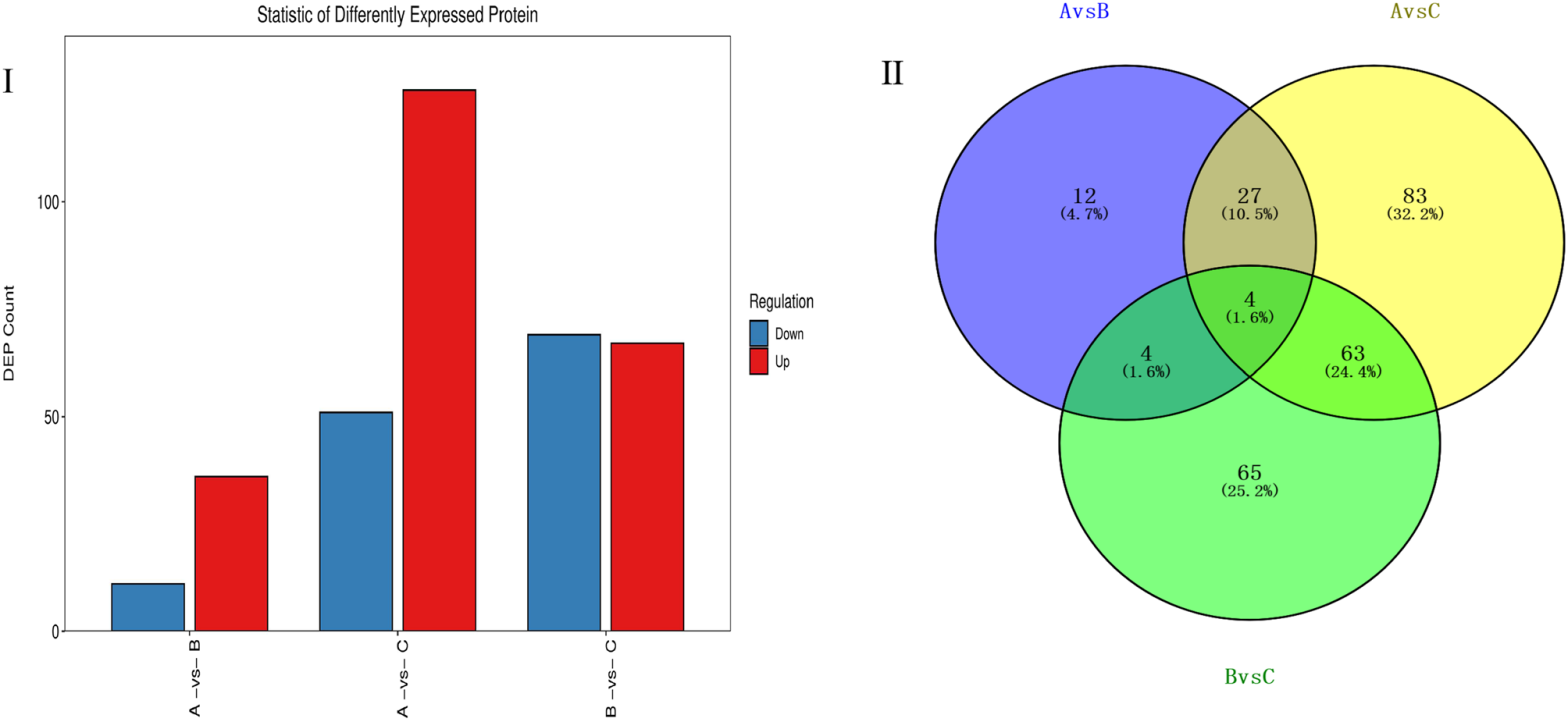
Number of differentially expressed proteins (DEPs) and their Venn diagram analysis. *Note*: A vs. B denotes B compared with A; likewise, A vs. C denotes C compared with A, and B vs. C is C compared with B. The same for figures below.

**Figure 3:**
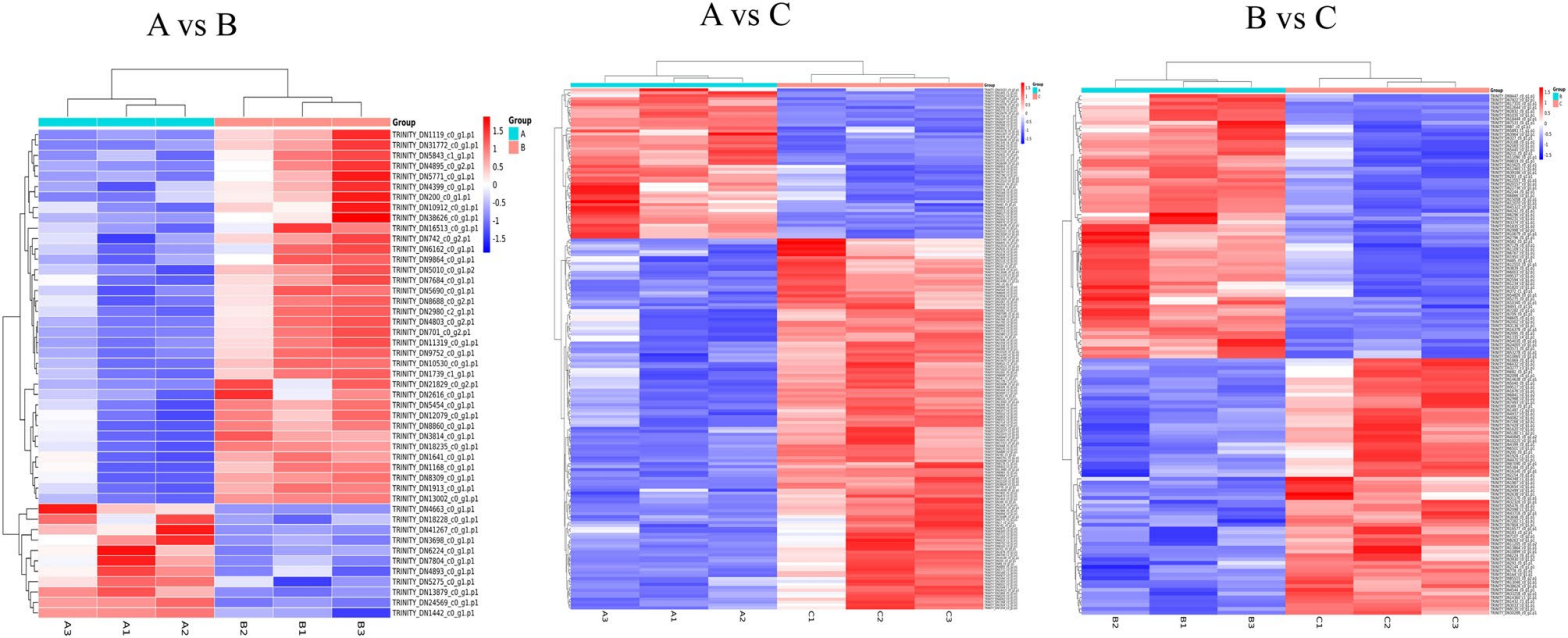
Hierarchical clustering analysis for differentially expressed proteins under salt stress. *Note*: Blue shading reflects the degree of decrease in protein expression, while red shading reflects the degree of increase in protein expression.

**Figure 4:**
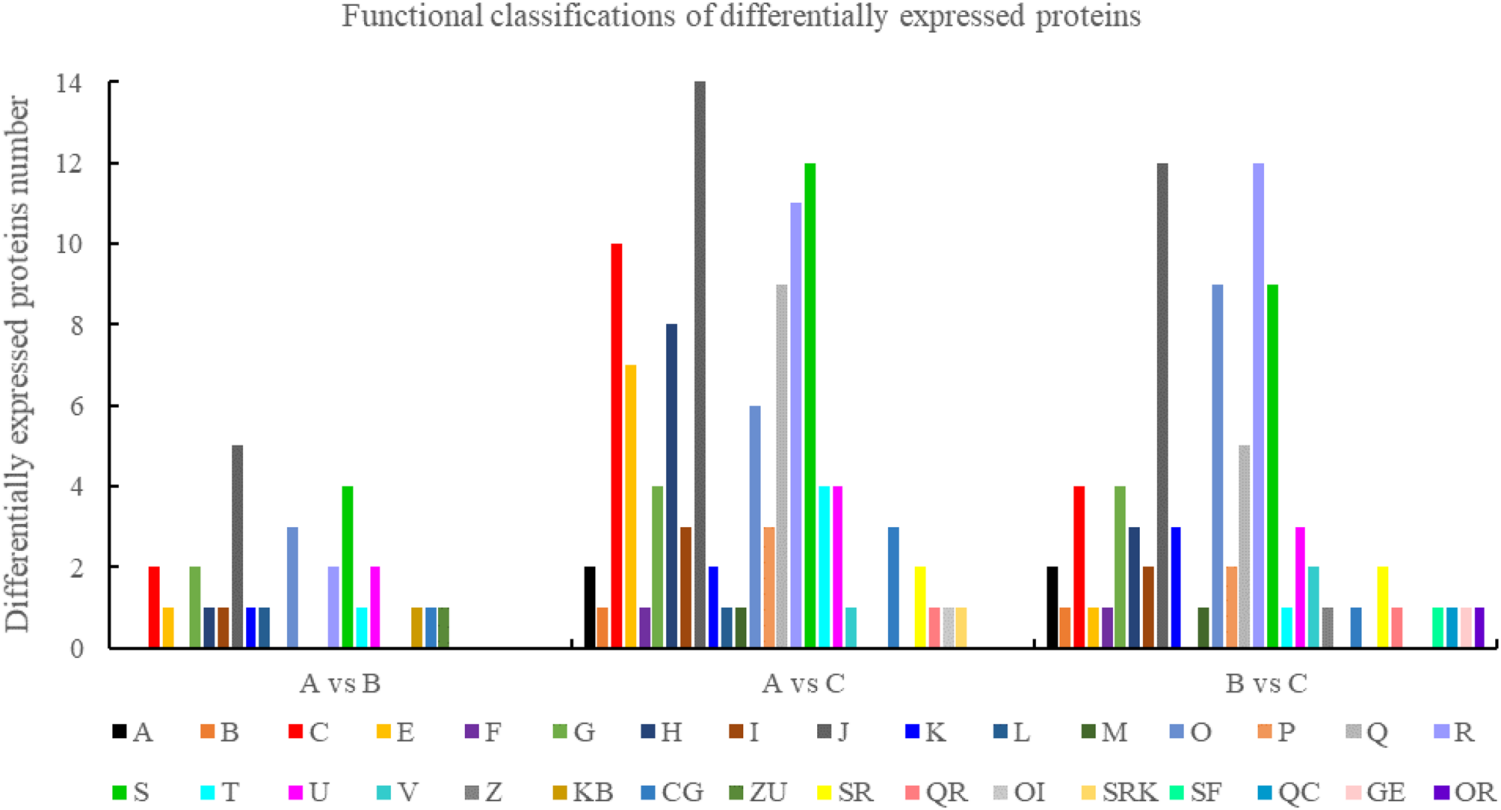
Functional classifications of differentially expressed proteins. *Note*: respectively. A: RNA processing and modification; B: Chromatin structure and dynamics; C: Energy production and conversion; E: Amino acid transport and metabolism; F: Nucleotide transport and metabolism; G: Carbohydrate transport and metabolism; H: Coenzyme transport and metabolism; I: Lipid transport and metabolism; J: Translation, ribosomal structure and biogenesis; K: Transcription; L: Replication, recombination and repair; M: Cell wall/membrane/envelope biogenesis; O: Posttranslational modification, protein turnover, chaperones; P: Inorganic ion transport and metabolism; Q: Secondary metabolites biosynthesis, transport and catabolism; R: General function prediction only; S: Function unknown; T: Signal transduction mechanisms; U: Intracellular trafficking, secretion, and vesicular transport; V: Defense mechanisms; Z: Cytoskeleton;

**Figure 5:**
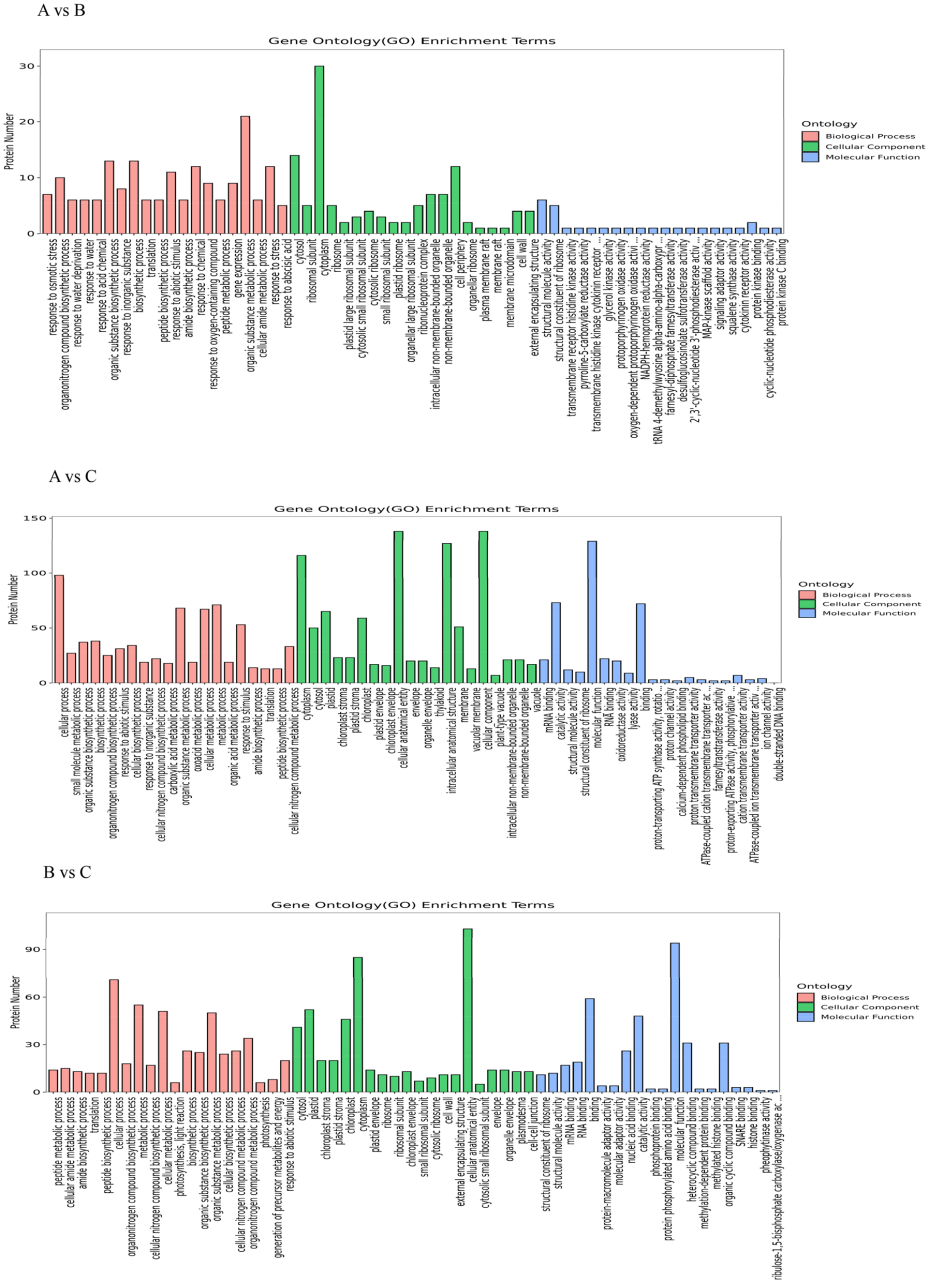
Gene Ontology (GO) annotation of differentially expressed proteins under the salt stress. *Note*: Enrichment results for the three categories are shown in the figure, with up to 20 items for each.

**Figure 6:**
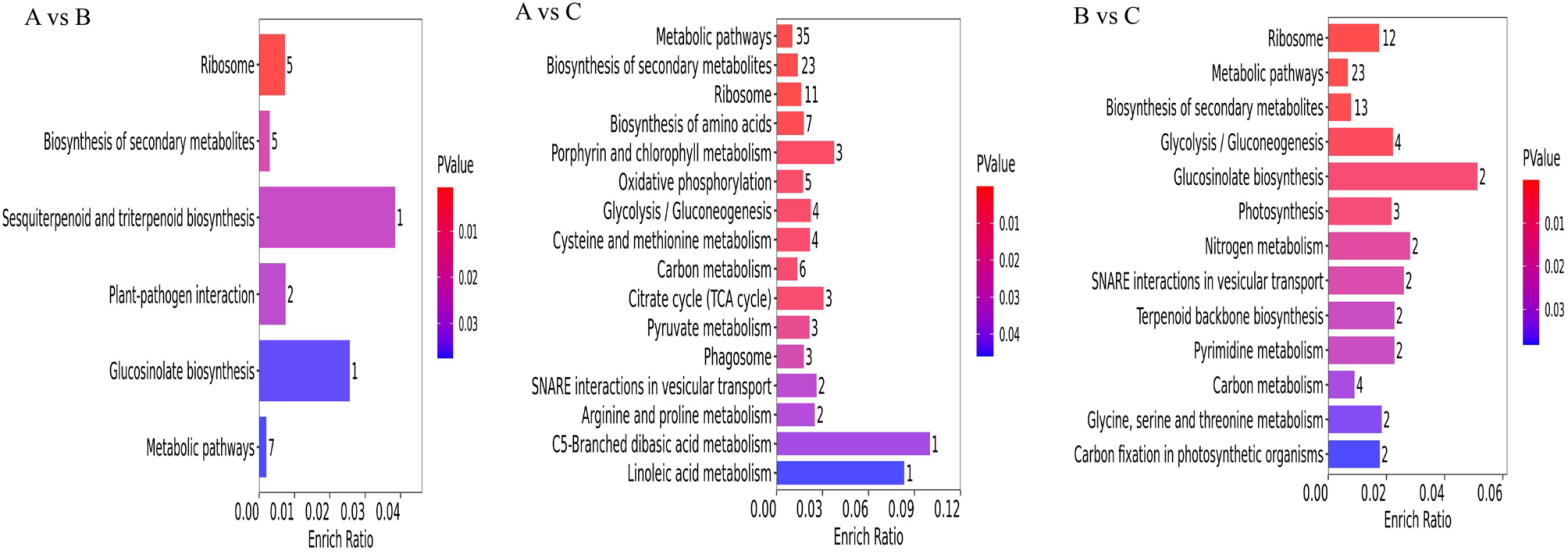
Bar diagrams of the differential proteins’ KEGG enrichment results.

**Figure 7:**
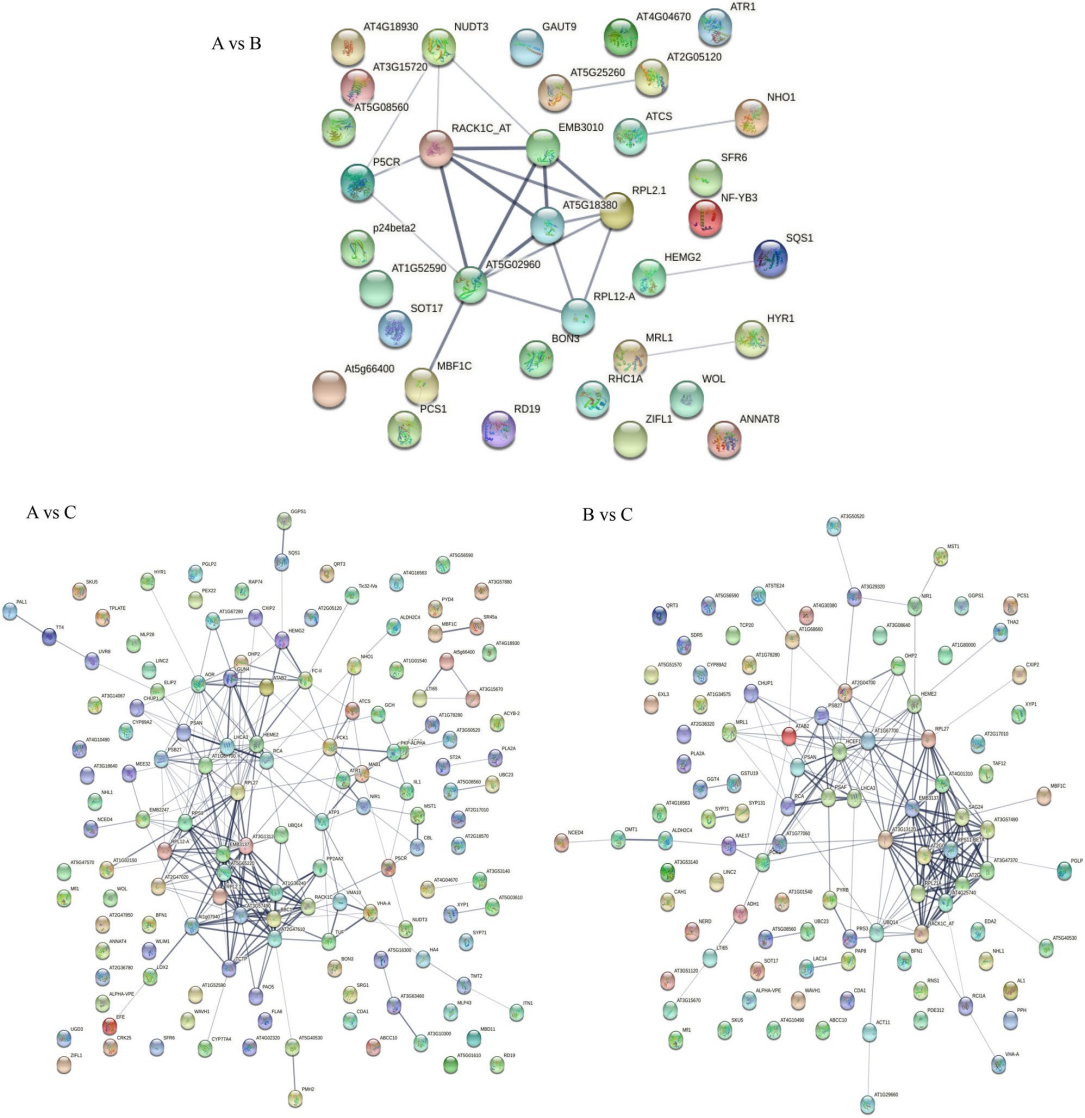
Protein interaction network diagram. *Note*: The "node" circles represent proteins. Different colors indicate different proteins. The straight line shows the interaction between proteins; the thicker the line, the stronger the interaction.

### Number of differentially expressed proteins (DEPs)

Compared with group A, 47 DEPs were obtained from group B, of which 36 proteins were up-regulated and 11 proteins were down-regulated. Compared with group A, 177 DEPs were obtained from group C, with 126 of them up-regulated and the other 51 proteins down-regulated. Compared with group B, 136 DEPs were obtained from group C: 67 and 69 that were up- and down-regulated, respectively (Fig. 2-I). The identified proteins with significantly different expressions were statistically analyzed, and a certain number of proteins were found common among the three groups, as depicted in Fig. 2-II. Evidently, different proteins appeared in the three groups, and there were 12, 83, and 65 specific proteins in the A vs. B, A vs. C, and B vs. C comparison groups, respectively. In both A vs. B and A vs. C groups, 31 differential protein sites were found. Among these, 27 differential protein sites were up-regulated and 4 were down-regulated. In the A vs. B and B vs. C groups, there were 8 differential protein sites, 3 up-regulated and 1 down-regulated, while the other four DEPs showed opposite expression patterns in the two comparison groups. There were 67 differential protein sites in both A vs. C and B vs. C groups: 37 were up-regulated and 30 down-regulated. These results indicated significant differences in protein expression occurred between low salt (B) and high salt (C) conditions in *R. soongorica* seedlings. Notably, 83 proteins were only expressed in A vs. C under high salt stress, and this number significantly exceeded that of other comparison groups. This may point to the self-protection of plants under high salt stress by initiating greater levels of gene expression.

### Hierarchical clustering analysis for DEPs under salt stress

As seen in Fig. 3, each column in the graphic represents a sample, and each row represents a protein; color represents the relative expression level of a given protein in the group of samples. On the left is the tree of protein clustering: the closer the branches of two proteins are, the closer their expression levels are, namely, the closer the trends in their variation. By analyzing the up-regulation and down-regulation of different proteins in different sample groups, we can tell that the similarity between the three repeated samples in each group is very high, which would support screening the DEPs accordingly.

### Functional classification of DEPs according to the Clusters of Orthologous Groups (COGs) under salt stress

Figure 4 shows that under the salt stress response, DEPs are involved in different biological processes. These included RNA processing and modification, chromatin structure and dynamics, energy production and conversion, amino acid transport, nucleotide transport, and metabolism, among others. There were 29 differential proteins in the A vs. B comparison group that could be annotated and functionally classified by the COG database. These differential proteins were mainly involved in translation, ribosomal structure and biogenesis, function unknown, post-translational modification, protein turnover, and chaperones biological process, of which 21 were up-regulated and 8 were down-regulated. In the A vs. C comparison group, 112 differential proteins were annotated and functionally classified by COG database, these chiefly involved in translation, ribosomal structure and biogenesis, function unknown, general function prediction only, biological process of energy production and conversion, of which 78 and 34 respectively were up- and down-regulated. There were 86 differential proteins found in the B vs. C comparison group that could be annotated and functionally classified by the COG database. They were mostly involved in translation, ribosomal structure and biogenesis, general function prediction only, post-translational modification, protein turnover, and chaperones biological process, with 35 up-regulated and 51 down-regulated.

### Gene ontology (GO) enrichment analysis for DEPs under salt stress

After their GO annotation, differential proteins were classified according to the functional categories of molecular function (MF), cell component (CC), and biological process (BP). Major biological functions performed by the DEPS could be determined by a GO significance analysis. In the A vs. B control group analysis, 276 GO items were obtained (P < 0.05), consisting of 194 BP items, 31 CC items, and 51 MF items, with 14 differential proteins annotated by GO. These DEPs were mainly enriched in translation, response to external stimulus, intracellular structure, ribosome and structural constituent of ribosome, and metabolic process, etc. In the A vs. C control group analysis, 495 GO items were obtained (P < 0.05), namely 274 BP items, 88 CC items, and 133 MF items, with 82 differential proteins annotated by GO. These DEPs were mainly enriched in translation, biosynthetic process, ribosome, membrane, structural constituent of ribosome, oxidoreductase activity, and in other ways. In the B vs. C control group analysis, 390 GO items were obtained (P < 0.05), comprising 213 BP items, 78 CC items, and 99 MF items, with 50 differential proteins annotated by GO. These DEPs were mainly enriched in translation, photosynthesis, cytoplasm, small ribosomal subunit, structural constituent of ribosome, and RNA binding, etc. (Fig. 5).

### KEGG pathway enrichment analysis of DEPs

In the face of salt stress, protein functioning depends on the synergistic action of multiple proteins, resulting in significant changes in terms of their abundance. Pathway analysis can provide a more comprehensive and systematic understanding of the biological process each protein is relevant to, and thus point to and reveal the metabolic network of salt stress. In order to further understand the biological functions of the uncovered DEPs, their KEGG enrichment analysis was performed. These results showed that (Fig. 6) in the A vs. B comparison group, differential proteins were significantly enriched (P < 0.05) to six metabolic pathways (sesquiterpenoid and triterpenoid biosynthesis, glucosinolate biosynthesis, plant–pathogen interaction, ribosome, etc.). The differential proteins of A vs. C comparison group were significantly enriched to 16 metabolic pathways (linoleic acid metabolism, C5-dibasic acid metabolism, porphyrin and chlorophyll metabolism, etc.). Finally, the differential proteins in the B vs. C comparison group were significantly enriched to 13 metabolic pathways (glucosinolate biosynthesis, nitrogen metabolism, SNARE interactions in vesicular transport, etc.).

### Identification of protein–protein interaction (PPI) networks among DEPs

To investigate the biological function and regulation of DEPs in *R. soongorica* leaves under salt stress, and to uncover those key proteins related to salt tolerance. For this, a composite score of PPIs (protein interactions) greater than 0.4 was used to determine the interaction network. As Fig. 7 shows, five histone interactions were identified in the A vs. B comparison group, and a total of 17 DEPs were involved in the protein interaction network of seedlings under salt stress. The RPS23B (40S ribosomal protein S23-2) and RACK1C (receptor for activated C kinase 1C) had 7 and 6 node connections, respectively. The node connections of AT5G18380 (40S ribosomal protein S16-3), RPL2-A (50S ribosomal protein L2), and EMB3010 (40S ribosomal protein S6-2) numbered 5 in each case. For P5CR (pyrroline-5-carboxylate reductase), NUDT3 (nudix hydrolase 3), and RPL12-A (60S ribosomal protein L12), each had 3 node connections. In the A vs. C comparison group, 87 DEPs were involved in the protein interaction network under salt stress, and 18 of them had more than 10 node connections, with the most node lines obtained for RPS13 (30S ribosomal protein S13, 21), RPS10 (30S ribosomal protein S10, 21), RPL27 (50S ribosomal protein L27, 17), PPOX2 (protoporphyrinogen oxidase 2, 17), RPL29 (50S ribosomal protein L29, 14), and RPL2-A (50S ribosomal protein L2, 14), etc. In the B vs. C comparison group, 61 DEPs were involved in the protein interaction network under salt stress; 14 of them had more than 10 node connections, with the most node lines found for RPS13 (30S ribosomal protein S13, 20), CFBP1 (fructose-1,6-bisphosphatase 1, 18), RPS10 (30S ribosomal protein S10, 17), RPL27 (50S ribosomal protein L27, 15), RPS11C (40S ribosomal protein S11-3, 14), and RPL5 (50S ribosomal protein L5, 13), etc.

### Screening of key DEPs in leaves of *R. soongorica* seedlings under salt stress

Based on the COG database, 29, 112, and 86 DEPs in A vs. B, A vs. C, and B vs. C comparison groups could be annotated and functionally classified. Combined with the differential protein interaction regulatory network, protein points with a node connection number > 1 were selected and repeated proteins in each comparison group were integrated to further screen out the 72 DEPs. The abundance of 34 DEPs increased and 38 DEPs decreased under the salt stress treatments. The most varied proteins were involved in translation, ribosomal structure and biogenesis, amounting to 20 of them, of which 17 belonged to the ribosomal protein family (RP). Six ribosomal proteins (RPL2-A, RPL12A, RPS23B, RPS6B, RPL30A, and RPS16C) were up-regulated, while the expression levels of another 11 (RPL5, RPS13, RPS10, RPS2D, RPS11C, RPS20B, RPL21A, RPL27, RPS9, RPL29, and RPL7AA) were down-regulated under the NaCl stress. These results showed that *R. soongorica* seedlings could tolerate stress by synthesizing and degrading proteins in response to salt stress conditions. Furthermore, some proteins (GUN4, MRL1) with pronounced expression differences but not any reported functions were also found. These will be investigated in planned future work. The score of each protein was calculated by Mascot search software^22^. If the score was more than 60, the protein was considered reliable. Finally, four categories of concern were determined and their related DEPs artificially grouped: those proteins related to plant energy and metabolism, those proteins associated with photosynthesis, those proteins related to plant defense and stress resistance, and those participating in protein synthesis, processing, and degradation (Table 2).

**Table 2:** The key DEPs significantly expressed under salt stress.

Accession number	Gene name	Fold change	P value		Up/down	Comparison group	Protein score	Protein name
**Proteins related to plant energy and metabolism**								
O23654	*VHA-A*	1.513	0.006		up	A vs C	78.45	V-type proton ATPase catalytic subunit A
		1.692	0.002		up	B vs C	78.45	
P20115	*CSY4*	2.348	0.019		up	A vs B	89.41	Citrate synthase 4, mitochondrial
		3.893	2.563*10^–4^		up	A vs C	89.41	
Q39258	*VHA-E1*	1.690	0.029		up	A vs C	73.58	V-type proton ATPase subunit E1
Q9SU58	*AHA4*	1.538	1.862*10^–4^		up	A vs C	134.93	ATPase 4, plasma membrane-type
Q9T074	*PCK1*	1.746	8.648*10^–4^		up	A vs C	99.35	Phosphoenolpyruvate carboxykinase (ATP) 1
		1.868	0.002		up	B vs C	99.35	
**Proteins associated with photosynthesis**								
P10896	*RCA*	0.571	0.001		down	A vs C	61.72	Ribulose bisphosphate carboxylase /oxygenase activase, chloroplastic
		0.583	0.001		down	B vs C	61.72	
Q9SHE8	*PSAF*	0.562	0.024		down	B vs C	76.16	Photosystem I reaction center subunit III, chloroplastic
P49107	*PSAN*	0.590	0.004		down	A vs C	65.55	Photosystem I reaction center subunit N, chloroplastic
		0.546	0.012		down	B vs C	65.55	
Q9LR64	*PSB27-1*	0.614	0.001		down	A vs C	85.30	Photosystem II repair protein PSB27-H1, chloroplastic
		0.650	0.005		down	B vs C	85.30	
**Proteins related to plant defense and stress tolerance**								
P54904	*P5CR*	1.515	0.006		up	A vs B	63.85	Pyrroline-5-carboxylate reductase
		3.112	0.003		up	A vs C	63.85	
O04921	*FC2*	1.675	0.028		up	A vs C	95.80	Ferrochelatase-2, chloroplastic
Q9SF29	*SYP71*	1.619	0.015		up	B vs C	88.56	Syntaxin-71
Q9SRV7	*SYP131*	1.673	0.016		up	B vs C	74.66	Putative syntaxin-131
**Protein synthesis, processing and degradation**								
P56791	*rpl2-A*	2.096	0.011		up	A vs B	60.50	50S ribosomal protein L2, chloroplastic
		2.892	0.052		up	A vs C	60.50	
P36210	*RPL12A*	2.832	0.009		up	A vs B	71.98	50S ribosomal protein L12-1, chloroplastic
		2.543	0.013		up	A vs C	71.98	
P51430	*RPS6B*	1.909	0.038		up	A vs B	61.36	40S ribosomal protein S6-2
P49692	*RPL7AA*	0.651	0.032		down	A vs C	63.75	60S ribosomal protein L7a-1
Q9SCM3	*RPS2D*	0.579	7.254*10^–5^		down	A vs C	60.24	40S ribosomal protein S2-4
Q9LK61	*RPS10*	0.659	0.021		down	A vs C	75.71	30S ribosomal protein S10, chloroplastic
		0.616	0.002		down	B vs C	75.71	
Q9XJ27	*RPS9*	0.642	2.142*10^–5^		down	A vs C	73.93	30S ribosomal protein S9, chloroplastic
A2RVR7	*At2g47020*	1.738	6.313*10^–4^		up	A vs C	95.71	Peptide chain release factor 1, mitochondrial

## Discussion

### Effect of NaCl concentration on growth indicators and leaf physiological indexes of *R. soongorica* seedlings

Salinity is undoubtedly a worsening worldwide problem, being a major abiotic stress affecting the growth, development, and productivity of plants^23^. Nevertheless, there are differences in the mechanisms of tolerance to salt stress among different species of plants. Osmotic stress is a direct response of plants to salt stress, and plants can mitigate their incurred damage by regulating intracellular regulatory substances^24,25^. In addition, the generation and transport of biomass is also an important factor in assessing the degree of salt stress in plants^26^. Studies have shown that salt stress reduces plant biomass synthesis, and that plant leaves are capable of responding to salt stress via the rapid accumulation of proline^27–29^. In our experiment, under the stress condition of low salt B (200 mM NaCl), the fresh weight and root/shoot ratio of *R. soongorica* seedlings increased, and low salt had a significant promotional effect on their growth. Yet when the NaCl concentration reached C (500 mM NaCl), the seedlings’ fresh weight and root-shoot ratio decreased, and their growth was inhibited significantly. These results, which are consistent with those of Nasim et al.^30^ for butterfly pea could be explained by the high salt tolerance of *R. soongorica* in the imposed salinity range; specifically, by it effectively relying on Na^+^ and Cl^−^ accumulation to regulate cell permeability and maintain expansion, and via effective K^+^ homeostasis to maintain stomatal functioning in leaves. The inhibition of seedling growth under high salt conditions is likely due to the limited ability to isolate Na^+^ and Cl^−^ in vacuoles^31^. The proline content in *R. soongorica* leaves increased significantly with a greater salt concentration, a result consistent with findings of previous studies, possibly because the proline synthesis gene was activated or the expression of proline degradation gene was inhibited under salt stress^28^.

### DEPs in leaves of *R. soongorica* under salt stress

#### Proteins related to plant energy and metabolism

It is necessary for plant growth and development to produce catabolic energy in the face of salinity. Glycolysis and the tricarboxylic acid (TCA) cycle are the major pathways for energy production^32^, and CS is a key enzyme of the TCA cycle^33^. Up-regulation of CS expression can improve the tolerance of maize to salt stress^34^. We found that the expression of the CS family protein (CSY4) was up-regulated by 2.35 and 3.89 times under low salt (A vs. B) and high salt (A vs. C) stress, respectively. Up-regulation of the TCA pathway would contribute to the production of *R. soongorica* catabolic energy to support its seedling growth under salt stress conditions. V-ATPase plays a key role in activating the secondary active transport of plants and is an indispensable enzyme in plants, especially for coping with abiotic stresses^35^. For example, overexpression of the vacuolar V-ATPase C subunit protein gene was shown to augment the salt tolerance ability of tobacco^36^. We found two isoforms (VHA-A, VHA-E1) of V-ATPase in leaves of *R. soongorica*. Both were up-regulated under imposed salt stress, while AHA4 was up-regulated in the plasma membrane. This may be because the up-regulated expression levels of VHA-A, VHA-E1, and AHA4 are associated with the uptake and transport of Ca^2+^ and K^+^, which are involved in the regulation of protein homeostasis under salt stress. Phosphoenolpyruvate carboxykinase (PCK1) functions as a catalytic enzyme, converting oxaloacetate—to regulate protein homeostasis under salt stress—into phosphoenolpyruvate, an intermediate product in glycolysis^37^. The increased expression of this protein in *R. soongorica* leaves under salt stress may be linked this plant’s ability to tolerate salt stress.

#### Proteins associated with photosynthesis

Photosynthesis is the most important process in plant metabolism^38^. Rubisco is a key enzyme in the dark reaction of photosynthesis and plays a central role in carbon fixation^39^. Under salt stress, rubisco protein’s expression in *Prunus mume* leaves was down-regulated^40^, but its expression in *Haloxylon salicornicum* leaves was up-regulated^41^. We found that rubisco (RCA) expression was down-regulated in the A vs. C and B vs. C comparison groups, suggesting that down-regulated rubisco expression may lead to reduced light energy utilization in *R. soongorica* leaves while under salt stress. In plants, PSAN is the subunit that mediates LHCII energy transfer to the photosystem I (PSI) core and it figures prominently in fostering efficient electron transport from plastocyanin to P700. In eukaryotic photosynthetic organisms, the PSI subunit PSAF is involved in the docking of the electron-donor proteins plastocyanin and cytochrome c6^42^; however, salt-alkali stress can significantly reduce the binding stability between the subunits of PSI^43^. In our study, the expression levels of both PSAN and PSAF in *R. soongorica* were decreased under salt stress, perhaps because salt stress inhibits the electron transfer mechanism in its leaves. Photosystem II (PSII) is prone to photoinduced damage; hence, it is continuously repaired to maintain its function. The Psb27 protein interacts with the CP43 subunit of PSII and participates in this repair of PSII, and in *Arabidopsis* two PSB27-like proteins (PSB27-H1 and PSB27-H2) were found involved in PSII repair^44^. Our study found that the PSB27-1 protein was down-regulated by 0.61 and 0.65 times in the A vs. C and B vs. C comparison groups, respectively, which could be explained by damage to both the donor side and recipient side of PSII, and severe photoinhibition of both PSII and PSI. These results suggest that these four photosynthesis-related proteins may play important roles in the leaf response to salt stress.

#### Proteins related to plant defense and stress tolerance

Pyrroline-5-carboxylic acid reductase (P5CR) is a terminal enzyme that functions critically in proline biosynthesis^45^. To improve their salt tolerance, the leaves of *Sorghum bicolor*^46^ and sweet potato^47^ can accumulate large amounts of proline via overexpression of the P5CR protein under salt stress. In our study, the P5CR protein was up-regulated by 1.51 and 3.11 times under low and high salt stress, respectively, which further explains why the proline content increased under salt stress as shown in Fig. 1-II. It has been suggested that proline could induce the expression of responsive genes in response to physiological stress caused by too much salinity. Previous reports have revealed that salt stress induced the expression of FC2 in *Arabidopsis* leaves^48^. Likewise, in *R. soongorica*, high salt stress caused the FC2 to increase by 1.68 times. This shows that under high salt conditions, FC2 protein expression can effectively control the transport and distribution of metal in the cell, enabling each leaf cell to reach a steady state equilibrium, which demonstrates a certain tolerance to salt stress. SNARE proteins drive vesicle transport and transport membrane functioning, taking cargo to target sites within and on the cell surface, thereby contributing to cell homeostasis, morphogenesis, and pathogen defense^49^. SYP proteins are a family of QC-SNARE proteins unique to plants. In *Arabidopsis*, SYP121 interacts with KAT1 and KC1 (K channels) to regulate K^+^ currents in the plasma membrane^50^. The up-regulated expression of SlSYP51.2 protein is known to enhance tomato plant’s tolerance of salt stress^51^. In our study, both SYP71 and SYP131 proteins were up-regulated under high salinity (A vs. C), suggesting these proteins may similarly regulate the activity of metal ion channels and thus improve *R. soongorica*’s tolerance of salt stress.

#### Protein synthesis, processing, and degradation

Ribosome synthesis can trigger the nucleolar stress response and activate p53, thus maintaining the stability of the intracellular environment^52^. The ribosome consists of two parts, the case ribosomal protein (RP) and ribosomal RNA. The RP not only maintains the configuration of RNA, but also participates in the synthesis, transport, and localization of proteins^53^. Salinity reduced the ribosomal protein expression abundance in creeping bentgrass^54^. Li et al.^55^ found that for upland cotton under salt stress, the abundance of two ribosomal proteins decreased whereas that of two ribosomal proteins increased. Our data showed the abundance of three ribosomal proteins *(*RPL2-A, RPL12A, and RPS6B) increasing significantly under low salt stress, which suggests the overall protein synthesis level of *R. soongorica* seedlings increased under conditions of low salt stress and promoted this plant’s growth. Under high salt stress, the abundance of two ribosomal proteins (RPL2-A and RPL12A) increased significantly, and the abundance of four ribosomal proteins (RPS10, RPS2D, RPS9 and RPL7AA) decreased significantly. Yet at the same time the expression of a peptide chain releasing factor (AT2G47020) was significantly up-regulated, indicating that the overall protein synthesis level of *R. soongorica* seedlings is decreased under conditions of high salt stress. The differential regulation of different ribosomal proteins in the translation mechanism suggests that *R. soongorica* seedlings cope with high salt stress by balancing ribosomal proteins’ synthesis and degradation.

## Materials and methods

### Plant materials

Seeds of *R. soongorica* were collected from Laohukou, Wuwei, in Gansu Province, China (102°58′E, 38°44′N; elevation 1315–1375 m), in late October 2019. The average annual temperature, rainfall, and evaporation of this sampling area is 7.5 °C, 110 mm, and 2,646 mm, respectively. The seeds (voucher numbers: 063–2) were identified by Dr. X. Liu, at the Institute of Gansu Minqin National Studies Station for Desert Steppe Ecosystems (MSDSE); seed samples were deposited at the Herbarium of Scientific Research Experimental Station of the Longqu Seed Orchard, Gansu Province Academy of Qilian Water Resource Conservation Forests Research, in Zhangye. These seeds were put inside a seed storage cabinet (CZ-250FC, Top Yunong, Zhejiang, China) until their later use. Plant materials were collected in strict accordance with the Technical Regulations for the seed Collection of Rare and Endangered wild Plants of the People’s Republic of China (LYT2590–2016).

### Plant growth and salt treatments

The experimental research on plants were carried out in accordance with technical regulations for cultivation of tree seedings (DB11T476-2007), Forestry Industry Standard (LY/T 1898–2010) and soil and Water conservation test Standard (SD 239–87) issued by the Ministry of Water Resources of the People's Republic of China. The experiments were carried out in the No. 4 experimental shed of the Scientific Research Experimental Station of Longqu Seed Orchard, in Zhangye, Gansu Province, China (100° 22'E, 38° 78'N; elevation 1591–1681 m). In April 2020, uniform full-sized seeds were selected and disinfected for 8 min with 1% NaClO and rinsed six times with ultra-pure water. Cleaned seeds were then sown in a plug tray (8.5 cm height × 4.5 cm diameter, with drainage holes at the bottom) filled with vegetative soil, quartz sand, and vermiculite (3:1:1), and sprinkler-irrigated with underground water. They were cultivated in a greenhouse at a temperature of 25 ± 1 °C under 50% humidity with ventilation and natural light. In July 2020, local farming soil was selected for use in the pot experiments; this soil type is that of irrigated desert soil. Before transplanting, an intelligent soil nutrient analyzer (TPY-6A, Top Yunong, Zhejiang, China) was used to determine the available phosphorus, ammonium nitrogen, salinity, and pH of the tested soil, which were 26.6 mg/kg, 10.0 mg, 0.2%, and 8.3, respectively. Next, uniformly germinated seedlings were transferred into plastic pots containing 2.5 kg of soil (pot dimensions: 23 cm wide at the top, 13 cm wide at the bottom, and 14 cm in height). Intelligent watering control systems were used to maintain the soil water content close to field capacity (i.e., 60%). The salt treatments were applied after 1 month of slow seedling. Seedlings were thinned to four plants per pot, with five pots in each group to which 0 (control, A), 200 (low salt, B), or 500 (high salt, C) mM NaCl was supplied, for a total of three treatment groups (15 pots, 60 seedlings in all). To reduce measurement error, each group was tested three times (Table 3). According to the experimental design, the corresponding NaCl solution was prepared with deionized water, and using a syringe it was evenly poured around the root system of *R. soongorica* plants. To avoid osmotic shock in seedlings caused by a salt shock reaction, the target concentration was reached over a 24-h-period via gradual salt applications. The NaCl treatment for 24–48 h was set as the NaCl treatment at day 0, and the relevant indexes were measured after 3 days.

**Table 3:** Experimental treatment.

Treatment	NaCl concentration		
	0 mM L^−1^(A)	200 mM L^−1^(B)	500 mM L^−1^ (C)
Group 1	A1	B1	C1
Group 2	A2	B2	C2
Group 3	A3	B3	C3

### Morphological and physiological indexes determination

#### Determination of plants’ wet weight, dry weight, and ratio of roots to shoots

The 10 plants from each treatment group were washed with distilled water and dried with absorbent paper. We then counted their main stem, branches, and leaves above the rhizosphere, as the above-ground plant parts. The fresh weight (FW) of roots and above-ground parts of the plants was obtained and their mean values per plant calculated. Then they were dried—at 105 °C in an oven for 30 min, and dried again to constant weight at 80 °C—and reweighed. We calculated their root/shoot ratio (R/T) = dry weight of underground part/dry weight of above-ground parts.

#### Measurement of a leaf’s total surface area

The total surface area of single plant leaf was measured with a leaf area analyzer (LI-3000C, Legol Tech, Beijing, China). The area of two clean plastic sheets was first measured to eliminate error. The *R. soongorica* leaves were removed with scissors and tweezers, and individually placed between the two plastic sheets and passed under the scanning head, to obtain their total surface area.

#### Determination of leaf relative conductivity and proline content

The relative conductivity of each sample was measured according to Hu et al.’s^56^ measurement method, with some modifications applied. We weighed 0.3 g of *R. soongorica* fresh leaves, rinsed off any surface stains, dried them with absorbent paper, placed them in a 50 ml conical bottle, added 20 ml of deionized water, and placed them at room temperature for 3 h, next we measured the conductivity of the solution with a conductivity meter (DDS-W, Bant instrument, Shanghai, China), recorded that, then put them into a thermostatic water bath at 100 °C, and let it boil for 15 min, cool down, and finally determined the conductivity of the solution as R2. The relative conductivity (%) in leaves was calculated as conductivity/the final conductivity × 100%. The proline content of the *R. soongorica* leaves was determined as described by Sajid et al.^57^.

### Analysis and identification methods of differential proteomics in leaves of *R. soongorica* under salt stress

The differential proteomics analysis and protein identification conducted for leaves of *R. soongorica* followed the described methods of Kumaravel et al.^58^, Holáet et al.^59^, and Kumar et al.^60^, with some modifications introduced. The preparation of proteome libraries and their deep sequencing were both performed by the Naomi Metabolic Technology Corporation (Suzhou, China).

#### Protein extraction and quantification

For this, a given leaf sample was taken from ultra-low temperature refrigerator (–80 °C), ground it into fine powder in liquid nitrogen at low temperature, and put it into an EP tube. Then a 100-μL powder subsample was added into a new EP tube, to which 500 μL methanol was added, mixed well, the allowed to rest on ice for 10 min, after which it was centrifuged (14 000 × *g*, 4 °C) for 5 min and the ensuing supernatant removed (repeated four times). Finally, the methanol residue in the precipitate was cleaned with 1 × PBS, centrifuged (14 000 × *g*, 4 °C) for 5 min, and the precipitate then collected. To each sample, 500 μL of an 8 M-urea lysis buffer was added, and this ultrasonicated on ice for 10 min (power: 15%, ultrasonic 3 s, stop 3 s). The protein was quantified by SDS-PAGE electrophoresis.

#### Trypsin digestion

According to the quantitative results of the electrophoretic diagram, 200 μL of protein lysate was taken from each sample and placed in a centrifugal tube for an enzyme digestion. We added 2 μL of 1 M DTT (DL-Dithiothreitol, Promega, Beijing, China) to each tube, mixed it, heated it at 56 °C for 15 min, and the briefly spun it by centrifuging, after which it was cooled to room temperature. Each sample was divided into four tubes: 50 μL per tube, to which 150 μL of 50 mM ABC (Vectastain ABC Kit, Jinpan, Shanghai, China) was added to attain a 2 M-urea concentration. Next, we added 1.5 μg trypsin (Putai, Hangzhou, China) to each tube, mixed it with a pipette gun, cut by enzymatic at 37 °C for 4 h, then added 1.5 μg trypsin, cut by enzymatic at 37 °C overnight. After this enzyme digestion process, 20 μL 10% FA (Fisher Scientific, A117-50, Fisher, America) was added and centrifuged at 14 000 × *g*, from which supernatant was removed for desalting. Finally, one sample was taken from each group, and each analyzed by liquid chromatography-tandem mass spectrometry for 1 h.

#### Peptide fragment labeling and fractionation

The labeled peptide was dissolved in a 0.1 M TEAB (triethylamine-carbonate) buffer (pH = 8.5). We took out a tube containing 0.1 mg of the TMT (TMT10plex™, Thermo Scientific™, America) reagent, added 12 μL of acetonitrile, vortexed it for 10 s, then placed it at room temperature for 5 min, and the repeated the vortex (3 times) to ensure the TMT reagent was fully mixed in. Then 10 μL of the peptide (10 μg) was removed and added to the corresponding TMT reagent, according to labeling information table (Table 4). Different samples were labeled with a different labeling reagent, and these thoroughly mixed by vortexing, and allowed to sit at room temperature for 1 h. After that, the labeling reaction was stopped. A 1-μg sample was taken from this mixture and mixed with 150 μL of 1% FA for desalting. The mass spectrometry method was used for detection, for which the labeling efficiency must be higher than 95% to reach the standard. The remaining mixture samples were stored at –80 °C to be separated. The eluent was mixed into two fractions by the strong cation exchange (SCX) method, and then the two fractions were added to different C18 reversed-phase columns. The peptides were eluted by an 80-μL CAN (cerium ammonium nitrate, Fisher Scientific, Pittsburgh, America) solution. Finally, the eluted peptides were mixed into six fractions and stored at –80 °C for the mass spectrometry detection.

**Table 4:** Peptide labeling information table.

TMT 126	TMT 127 N	TMT 127C	TMT 128 N	TMT 128C	TMT 129 N	TMT 129C	TMT 130 N	TMT 130C
A1	A2	A3	B1	B2	B3	C1	C2	C3

#### Mass spectrometry analysis

Orbitrap Fusion Lumos (Thermo Fisher Scientific, USA) mass spectrometer was used for the data collection. The ion source was nano current electrospray ion source (NSI) with a spray voltage of 2200 V and an ion transport capillary temperature of 320 °C. The mass spectrometry data were collected in the positive ion mode by data-dependent acquisition mode (DDA). Orbitrap was used for full scans at level 1. Secondary mass spectrometry acquisition was performed by fragmentation of parent ions with 38% high-energy collision dissociation (HCD), and the ensuing fragment ions were detected in Orbitrap.

#### Qualitative and quantitative analysis of proteins

After the completion of MS scanning, the total ion flow chromatogram of MS signal was obtained. After the mass spectrum data were inputted into Proteome Discoverer software (PD) (v2.2, Thermo Fisher Scientific), the software first screened the mass spectrum. This mass spectrum data was searched via the Sequest operation program embedded in the PD software. The same software conducted a qualitative analysis according to the Sequest search results and the spectrum (after the first screening step). By extracting the signal value of TMT reporting ions, the protein quantification value was recalculated, this represented here by its median, where the protein quantification value was the sum of the obtained peptide quantification values.

### Statistical and bioinformatics analysis

Microsoft Excel 2016 was used for data processing, and Origin 8.0 software was used for plotting. SPSS 22.0 analysis software was used for analysis of variance (ANOVA). The functional enrichment database did not provide the enrichment analysis of *R. soongorica* leaves. Accordingly, the identified protein sequences were compared with the background libraries of GO and KEGG via BLAST searches. For their relative quantification, protein expression abundance was set to determine the statistical significance of the difference and accurately identify the DEPs induced by salt stress. When the protein difference multiple was > 1.5 and its P-value < 0.05, it was considered an up-regulated protein; when the difference multiple was < 0.66 and the P-value < 0.05, the protein was designated as down-regulated. The functions of all identified proteins were determined by searching GO (Gene Ontology) analysis in UniProt database (http://www.uniprot.org), and proteins were classified according to their main functions. We used the string-DB (http://string-db.org/) protein interaction database (selecting *Arabidopsis thaliana*) to analyze the interaction of the compared and differentially expressed proteins.

### Ethics approval and consent to participate

The experimental research and field studies on plants or seeds in this work comply with the IUCN Policy Statement on Research Involving Species at Risk of Extinction and the Convention on the Trade in Endangered Species of Wild Fauna and Flora.

## Conclusion

As a salt-secreting plant, *R. soongorica* underwent a series of changes in its growth and differentially expressed proteins in its leaves while under controlled salt stress conditions. In terms of its growth indexes, low salt (200 mM·L^-1^) significantly promoted the vegetative growth (total leaf area, total fresh weight, root shoot ratio) of *R. soongorica* seedlings, while increasing the proline content of their leaves. Proteomic analysis revealed that energy- and metabolism-related proteins (P5CR, CSY4) and ribosomal proteins (RPL2-A, RPL12A, RPS6B) were up-regulated under low salt stress. However, the growth of *R. soongorica* seedlings was significantly inhibited under high salinity (500 mM·L^-1^). The reason for this may be that high salt decreases the abundance of proteins associated with photosynthesis (RCA, PSAF, PSAN, PSB27-1), ribosomal proteins (RPS10, RPS2D, RPS9, RPL7AA) yet it increases the abundance of a peptide chain-releasing factor (AT2G47020). Meanwhile, *R. soongorica* may respond to a high salt stress environment by up-regulating the expression of proteins related to energy and metabolism (VHA-A, VHA-E1, AHA4, CSY4, PCK1), defense and anti-stress related proteins (P5CR, FC2, SYP71, SYP131) and ribosomal proteins (RPL2-A, RPL12A) in its leaves. These proteins play an important role as a potential target protein conferring the salt tolerance ability of *R. soongorica*. This study lays a foundation for better understanding the molecular regulation mechanism underlying the salt stress response of *R. soongorica*.
