# Author Correction: Physiological response and proteomics analysis of *Reaumuria soongorica* under salt stress

**DOI:** 10.1038/s41598-022-26527-x

**Published:** 2022-12-19

**Authors:** Shipeng Yan, Peifang Chong, Ming Zhao, Hongmei Liu

**Affiliations:** 1 College of Forestry, Gansu Agricultural University, Lanzhou, 730070 China; 2 Gansu Province Academy of Qilian Water Resource Conservation Forests Research Institute, Zhangye, 734000 China

Correction to: *Scientific Reports* 10.1038/s41598-022-06502-2, published online 15 February 2022

The original version of this Article contained an error in the Acknowledgements section.

“The work was supported by the Regular Science and Technology Assistance Programs to Developing Countries (KY202002011); the Central Finance of Forestry Science and Technology Promotion Demonstration Funds of China (2020ZYTG15); the Natural Science Foundation of Gansu Province (20JR5RA035); the Science and Technology Innovation Base and Talents Program of Gansu Province (17JR7WA018); the National Natural Science Foundation of China (32160497); and the Physiological Characteristics and Molecular mechanism of Salt Tolerance of Reaumuria reaumuria in Response to Salt Stress (GAU-QDFC-2021-10).”

now reads:

“The work was supported by the Regular Science and Technology Assistance Programs to Developing Countries (KY202002011); the Central Finance of Forestry Science and Technology Promotion Demonstration Funds of China (2020ZYTG15); the Natural Science Foundation of Gansu Province (20JR5RA035); the Science and Technology Innovation Base and Talents Program of Gansu Province (17JR7WA018); the National Natural Science Foundation of China (32160407); and the Physiological Characteristics and Molecular mechanism of Salt Tolerance of Reaumuria in Response to Salt Stress (GAU-QDFC-2021-10).”

The original Article has been corrected.

